# Editorial

**Published:** 1879-08

**Authors:** 


### EDITORIAL.

WITH the present number, the BUFFALO MEDICAL AND SURGICAL JOURNAL enters upon a new volume, and is presented to the profession under a new management and in an improved dress. It is becoming to acknowledge that the high standing heretofore maintained by this JOURNAL, is due to the distinguished abilities of its past editors, Dr. Austin Flint, Sr., Dr. Austin Flint, Jr., Dr. Sanford E. Hunt, and Dr. Julius F. Miner; and with such a prestige, the new editorial staff makes its salutatory bow with the modesty becoming the debutante. The demands of the profession of to-day are fortunately so clear and well defined, that we have the plain duty before us of laboring with earnestness and energy to fulfill them satisfactorily to the numerous friends who have given the JOURNAL their constant support and sympathy in the past. We realize that a publication of this character is read mainly by busy practitioners in search of facts to be utilized and applied in the daily demands of their profession. Questions of a general or theoretical nature are of little interest to them, however attractive they may prove to the specialist. The first great aim, therefore, will be to give the JOURNAL an eminently practical character.

It is also anticipated that certain advantages will result from the plan of apportioning the labor into departments in charge of those qualified for the special duties assigned them. With this end in view, the work will be divided as follows:

Obstetrics and Diseases of Women and Children, by Dr. Lothrop.

Practice of Medicine and Pathology, by Dr. Van Peyma.

Chemistry, Materia Medica and Pharmacology, by Dr. Davidson.

Surgery, by Dr. Mynter.

Ophthalmology and Otology, by Dr. Howe.

This division of labor is a necessity demanded by the rapid advancement of medical science, and by the conviction continually forced upon the physician, that

> "One science only will one genius fit,
> So vast is art, so narrow human wit."

It is expected that the original articles will be in keeping with the high standard of literary and professional excellence that has characterized the past history of the JOURNAL. Its great advantage, however, will consist in condensed articles, culled from the most recent publications; nor will these be taken from English sources alone, but will embrace the latest records of medical and scientific research, translated from German, French, Dutch, and Scandinavian medical literature.

No labor will be spared to present in each monthly issue a digest of important facts, based on sound professional and ethical principles, which will aim to advance the interests of medical science, and fulfill the highest purpose of the medical press, to impart knowledge and disseminate information in the interests of humanity.

